# Zinner Syndrome: A Narrative Review of Imaging Findings with an Illustrative Case Report

**DOI:** 10.3390/diagnostics16081228

**Published:** 2026-04-20

**Authors:** Calin Schiau, Roxana Pintican, Simona Manole, Andrei Roman, Ioana Teofana Dulgheriu, Delia Doris Donci, Loredana Elisabeta Popa, Anca Ileana Ciurea, Ioana Bene

**Affiliations:** 1 Department of Radiology and Medical Imaging, Faculty of Medicine, “Iuliu Hatieganu” University of Medicine and Pharmacy, 400012 Cluj-Napoca, Romania; 2 Department of Radiology, Emergency Clinical County Hospital Cluj-Napoca, 400006 Cluj-Napoca, Romania; 3 Regina Maria Private Health Care Network, 400117 Cluj-Napoca, Romania; 4 Department of Radiology, “Prof. Dr. Ion Chiricuta” Institute of Oncology, 400015 Cluj-Napoca, Romania; 5 Department of Radiology, “Niculae Stancioiu” Heart Institute, 400001 Cluj-Napoca, Romania; 6 Department of Radiology and Medical Imaging, Nuclear Medicine, Faculty of Medicine, “Iuliu Hatieganu” University of Medicine and Pharmacy, 400012 Cluj-Napoca, Romania

**Keywords:** Zinner syndrome, seminal vesicle cyst, ejaculatory duct obstruction, renal agenesis, male pelvic cystic lesions, pelvic MRI, computed tomography, ultrasound and male infertility

## Abstract

Zinner syndrome is a rare congenital anomaly of the male genitourinary tract, characterized by the triad of unilateral renal agenesis, ipsilateral seminal vesicle cyst, and ejaculatory duct obstruction. Owing to its low prevalence and nonspecific clinical presentation, diagnosis is often delayed or incidental, with imaging playing a central role in detection and characterization. This study presents a narrative review with an illustrative case report, aiming to summarize the imaging features of Zinner syndrome, outline the main radiologic differential diagnoses of seminal vesicle cysts, and highlight common diagnostic pitfalls, with emphasis on cross-sectional imaging techniques such as computed tomography (CT) and magnetic resonance imaging (MRI). The narrative review of the literature highlights that CT and MRI are essential for accurate anatomical localization, characterization of cystic content, and identification of associated genitourinary anomalies. MRI, in particular, provides superior soft-tissue contrast and is considered the reference modality for diagnosis and differential evaluation of male pelvic cystic lesions. Key differential diagnoses include Müllerian duct cysts, prostatic utricle cysts, and ejaculatory duct cysts. As an illustrative example, we report the case of a young adult male presenting with pelvic discomfort, infertility, and mild lower urinary tract symptoms. Imaging findings, including ultrasound and cross-sectional studies, demonstrated a seminal vesicle cyst associated with ipsilateral renal agenesis, consistent with Zinner syndrome. Zinner syndrome should be considered in the evaluation of male pelvic cystic lesions, particularly in the presence of unilateral renal agenesis. Awareness of its characteristic imaging features is essential for accurate diagnosis and appropriate management, with MRI playing a pivotal role in confirming the diagnosis and distinguishing it from other pelvic cystic entities.

## 1. Introduction

### 1.1. Background

Congenital anomalies of the male genitourinary tract comprise a heterogeneous group of developmental disorders that may remain clinically silent or present later in life with nonspecific manifestations, including chronic pelvic pain, lower urinary tract symptoms, ejaculatory dysfunction, or infertility. Among these entities, Zinner syndrome is an exceptionally rare condition resulting from abnormal development of the Wolffian (mesonephric) duct. First described in 1914, it is classically defined by the triad of unilateral renal agenesis, ipsilateral seminal vesicle cyst, and ejaculatory duct obstruction [1,2].

Due to its low prevalence and variable clinical presentation, Zinner syndrome is frequently underdiagnosed or incidentally detected during imaging studies performed for unrelated indications. Clinical manifestations are often subtle and overlap with those of more common urologic or gastrointestinal disorders, thereby contributing to diagnostic delay [1,2,3]. In many cases, infertility or chronic pelvic discomfort in young adult males constitutes the initial clinical indication for further evaluation [1,4].

Imaging plays a central role in both detection and characterization. Ultrasound is typically used as the first-line imaging modality, particularly in the evaluation of infertility; however, its diagnostic performance is limited by operator dependence and suboptimal visualization of deep pelvic structures [5]. Computed tomography (CT) is valuable for confirming renal agenesis and providing a global anatomical overview; however, its relatively limited soft-tissue contrast reduces its ability to accurately characterize seminal vesicle lesions and ductal anatomy [6].

Magnetic resonance imaging (MRI) has therefore emerged as the reference standard, owing to its superior soft-tissue resolution, multiplanar capability, and detailed evaluation of the seminal vesicles, ejaculatory ducts, and associated mesonephric duct derivatives [5,6,7]. In addition, MRI enables characterization of cyst content and facilitates differentiation between congenital and acquired lesions, which is essential for establishing a confident diagnosis [7].

Despite these advantages, challenges remain in clinical practice. Seminal vesicle cysts may mimic a broad spectrum of pelvic cystic lesions, including Müllerian duct cysts, prostatic utricle cysts, ejaculatory duct cysts, and bladder diverticula, making differential diagnosis critical [5,8]. Furthermore, diverging views persist regarding the optimal imaging strategy. While CT is often sufficient to identify renal agenesis and may suggest the diagnosis, some authors consider it adequate for initial evaluation. In contrast, others emphasize that MRI is indispensable for definitive diagnosis, particularly for confirming lesion origin, assessing ejaculatory duct involvement, and characterizing atypical or complex cysts. This variability reflects a lack of standardized imaging pathways and contributes to potential misdiagnosis or incomplete evaluation [3,6].

Although awareness of Zinner syndrome has increased, the current literature is largely limited to isolated case reports and small case series. Comprehensive, radiology-focused reviews that integrate multimodality imaging findings with a structured diagnostic approach and detailed differential diagnosis remain relatively scarce, particularly in the recent literature [4,7].

### 1.2. Objectives

The present study aims to provide an updated, radiology-oriented review of Zinner syndrome, with emphasis on the role of ultrasound, CT, and MRI in diagnosis and evaluation. In addition to summarizing characteristic imaging findings, this review proposes a practical imaging-based diagnostic approach, highlights key elements for differential diagnosis of seminal vesicle cystic lesions, and discusses common interpretative pitfalls.

Furthermore, the manuscript includes an illustrative clinical case with atypical features, including associated ureteral anomalies, to expand the spectrum of imaging appearances. By integrating current evidence with practical radiologic guidance, this work aims to reinforce the central role of imaging—particularly MRI—in the accurate diagnosis and appropriate management of this rare condition.

## 2. Methods

This study was designed as a structured narrative review, conducted in accordance with the principles outlined in the SANRA (Scale for the Assessment of Narrative Review Articles) guidelines [9].

A comprehensive literature search was performed in PubMed, Scopus, Embase, the Cochrane Library, and Google Scholar, covering publications from January 2015 to December 2025. The final search was conducted in December 2025. Database-specific search strategies were adapted using combinations of Medical Subject Headings (MeSH) terms and free-text keywords, including: “Zinner syndrome”, “seminal vesicle cyst”, “ejaculatory duct obstruction”, “renal agenesis”, “male pelvic cystic lesions”, “pelvic MRI”, “computed tomography”, “ultrasound”, and “male infertility”. A representative PubMed search string was: (“Zinner syndrome” OR “seminal vesicle cyst”) AND (“MRI” OR “CT” OR “ultrasound” OR “imaging”). 

Eligible studies included original research articles, imaging-based case series, and clinically relevant case reports involving human subjects, published in English, and focusing on radiologic diagnosis, imaging findings, or image-guided management of Zinner syndrome. Exclusion criteria included: non-human studies, articles without imaging data, non-English publications, conference abstracts without full text, and studies lacking relevance to the imaging evaluation of the syndrome.

Titles and abstracts were independently screened by two reviewers, followed by full-text assessment of potentially eligible articles. Discrepancies were resolved through discussion and consensus. Reference lists of selected articles were manually screened to identify additional relevant studies.

The initial search yielded 48 articles. After removal of duplicates and application of inclusion and exclusion criteria, 26 articles were included in the final analysis. An additional 15 articles were identified through backward citation tracking.

Given the rarity of Zinner syndrome and the heterogeneity of available data, a narrative synthesis approach was adopted, focusing on embryology, clinical presentation, imaging characteristics, and differential diagnosis.

The case report included in this study was conducted in accordance with institutional ethical standards. Written informed consent was obtained from the patient for publication of clinical and imaging data.

## 3. Definition and Diagnostic Criteria

### 3.1. Definition

Zinner syndrome is an uncommon congenital anomaly of the male genitourinary system defined by the triad of unilateral renal agenesis, ipsilateral seminal vesicle cyst, and ejaculatory duct obstruction [10]. This syndrome arises from developmental abnormalities of the mesonephric (Wolffian) duct during early embryogenesis, leading to combined renal and lower genital tract anomalies [4,11]. Although historically considered rare, increasing use of cross-sectional imaging has led to more frequent recognition in clinical practice [12].

From a clinical standpoint, Zinner syndrome is regarded as the male counterpart of Mayer–Rokitansky–Küster–Hauser syndrome in females, as both disorders result from developmental abnormalities of the mesonephric or paramesonephric duct systems [13].

### 3.2. Diagnostic Criteria

The diagnosis of Zinner syndrome is primarily imaging-based and relies on the identification of its characteristic triad: (1) unilateral renal agenesis or severe ipsilateral renal dysgenesis, (2) a cystic lesion arising from the ipsilateral seminal vesicle, and (3) evidence of ejaculatory duct obstruction, which may be inferred from seminal vesicle enlargement, ductal dilatation, or associated infertility. While all three components support a definitive diagnosis, in clinical practice, the combination of seminal vesicle cyst and ipsilateral renal agenesis is often considered highly suggestive, particularly when confirmed on cross-sectional imaging.

MRI is regarded as the reference modality for establishing these criteria, as it allows accurate delineation of lesion origin, ductal anatomy, and associated anomalies, while also facilitating exclusion of alternative cystic pelvic lesions.

## 4. Embryologic Basis of Zinner Syndrome and Imaging Correlates

Zinner syndrome is a rare congenital anomaly of the male genitourinary tract resulting from aberrant development of the mesonephric (Wolffian) duct during the first trimester of gestation [14]. The mesonephric duct has a pivotal embryologic role, giving rise both to the ureteric bud—which subsequently forms the kidney and collecting system—and to ipsilateral reproductive structures, including the seminal vesicle, vas deferens, and ejaculatory duct. Consequently, defective ureteric bud development leads to ipsilateral renal agenesis, whereas abnormal distal mesonephric duct differentiation results in ejaculatory duct obstruction with secondary cystic dilatation of the seminal vesicle [11,15].

This shared embryologic origin underlies the classic imaging triad of Zinner syndrome: unilateral renal agenesis, ipsilateral seminal vesicle cyst, and ejaculatory duct obstruction. From a radiologic perspective, understanding this developmental relationship is fundamental, as it explains the consistent association of upper urinary tract and ipsilateral seminal vesicle abnormalities. The cystic component often becomes more conspicuous after puberty, when increased seminal vesicle secretory activity promotes progressive distension, making the anomaly more readily detectable on ultrasound, CT, or MRI [11,16].

Although only a few hundred cases have been described in the literature [7], recognition of its embryologic basis is essential for accurate imaging interpretation and for distinguishing Zinner syndrome from other retrovesical cystic lesions.

## 5. Genetics of Zinner Syndrome

The genetic basis of Zinner syndrome remains poorly defined, and current knowledge is based on limited evidence from isolated case reports and small sequencing studies [17,18]. While the syndrome is widely considered to result from aberrant embryologic development of the distal mesonephric (Wolffian) duct, a specific molecular driver has not been consistently identified. Preliminary reports using whole-exome sequencing have described candidate variants in individual cases; however, these findings remain hypothesis-generating and require validation in larger cohorts before any causal relationship can be established [19]. At present, the available data support an embryologic developmental anomaly rather than a confirmed monogenic disorder, and any proposed molecular mechanisms should therefore be interpreted with caution [20].

## 6. Imaging Diagnostics and Implications

Magnetic resonance imaging is subsequently employed for definitive lesion characterization and comprehensive pelvic anatomic mapping, owing to its superior soft-tissue contrast and multiplanar capabilities. This sequential approach facilitates accurate diagnosis and supports appropriate therapeutic planning.

### 6.1. Ultrasonography (US)

Ultrasonography is frequently the first imaging modality used in the evaluation of suspected Zinner syndrome because of its wide availability, portability, and noninvasive nature [21]. Transabdominal ultrasound may demonstrate an anechoic or complex retrovesical cystic lesion consistent with a seminal vesicle cyst, together with the absence of the ipsilateral kidney, thereby raising the initial suspicion of Zinner syndrome [4,11].

Transrectal ultrasonography can provide further delineation of seminal vesicle morphology and may better depict associated ejaculatory duct abnormalities. However, its diagnostic utility may be limited by operator dependence and patient tolerance [21].

Although ultrasonography is highly accessible and useful for initial assessment, its diagnostic performance may be limited by operator dependence, patient body habitus, and, in the case of transrectal imaging, patient tolerance. Nevertheless, it remains the preferred first-line modality in the diagnostic algorithm.

### 6.2. Computed Tomography (CT)

Computed tomography (CT) is generally employed as a second-line imaging modality in the diagnostic workup of Zinner syndrome. It is particularly useful when the anatomy of the urinary tract remains uncertain after ultrasonographic evaluation or when further assessment of the upper urinary system is required. In such cases, CT, including CT urography when indicated, can provide additional anatomic detail and improve visualization of the urinary tract.

CT reliably confirms ipsilateral renal agenesis and enables characterization of associated retrovesical cystic lesions, although its soft-tissue contrast resolution remains inferior to that of MRI. On CT images, the seminal vesicle cyst typically appears as a well-defined, low-attenuation lesion located posterior to the urinary bladder [4].

Overall, the role of CT is primarily complementary, particularly in patients with suspected renal anomalies, when MRI is not readily available, or in emergency settings that require rapid diagnostic evaluation [22].

### 6.3. Magnetic Resonance Imaging (MRI)

MRI is regarded as the gold standard for the detailed evaluation of Zinner syndrome owing to its superior soft-tissue contrast and multiplanar imaging capabilities [11,23]. MRI allows accurate and simultaneous assessment of seminal vesicle morphology, cystic dilatation, ejaculatory duct obstruction, ipsilateral renal agenesis or dysgenesis, and the relationship of the lesion to adjacent pelvic structures, including the prostate, urinary bladder, and rectum [22].

Compared with ultrasonography and CT, MRI provides more precise characterization of lesion origin, internal content, and ductal anatomy. This detailed assessment is essential for establishing a confident diagnosis, differentiating Zinner syndrome from potential mimics, and facilitating preoperative planning [11,24].

#### 6.3.1. Key Interpretation Considerations for Radiologists

Accurate interpretation requires a systematic assessment of associated findings. Particular attention should be given to the presence of ipsilateral renal agenesis, which is the most important discriminating feature.

The paramedian location of the cystic lesion is also essential, as midline lesions more commonly indicate alternative diagnoses. Demonstration of continuity with the seminal vesicle supports the lesion’s origin, while unilateral involvement further favors the diagnosis of Zinner syndrome.

Finally, caution is advised in lesion characterization, as most cases are benign; malignancy should only be suspected in the presence of solid enhancing components or clearly atypical features.

#### 6.3.2. Practical MRI Approach and Protocol

To facilitate a structured and practical imaging assessment, the key elements of MRI evaluation in Zinner syndrome are summarized in two complementary tables. Table 1 outlines a stepwise interpretative approach tailored for radiologists, emphasizing lesion identification, characterization, and recognition of associated anomalies. Table 2 presents a concise, clinically oriented MRI protocol, distinguishing between essential sequences and problem-solving acquisitions to optimize diagnostic accuracy while maintaining efficiency in routine practice.

## 7. Differential Diagnosis of Male Pelvic Cystic Lesions

The differential diagnosis of seminal vesicle cysts is broad and is based primarily on lesion location, morphology, and associated genitourinary anomalies (Table 3) [25]. Müllerian duct cysts typically present as midline retrovesical cystic lesions that do not communicate with the seminal vesicles and are not associated with renal agenesis. In contrast, prostatic utricle cysts are usually smaller midline lesions and are frequently associated with congenital genital anomalies, such as hypospadias [18].

Ejaculatory duct cysts may appear as paramedian cystic lesions and can closely mimic seminal vesicle cysts; however, they generally lack the characteristic association with ipsilateral renal agenesis observed in Zinner syndrome [5,18]. Ureteroceles, particularly ectopic variants, may also simulate seminal vesicle cysts on limited imaging studies and should be considered, especially when the distal ureteral anatomy is incompletely visualized [26].

Additional diagnostic considerations include pelvic abscesses, cystic neoplasms, and post-inflammatory or post-surgical cystic changes, which can usually be differentiated based on clinical context and specific imaging features [25].

Accurate radiologic characterization is essential, as several male pelvic cystic lesions may demonstrate overlapping imaging appearances and similar clinical presentations [27]. Among the available modalities, magnetic resonance imaging (MRI) is considered the imaging technique of choice because of its superior soft-tissue contrast resolution and multiplanar capabilities, allowing precise anatomical localization and detailed evaluation of associated genitourinary anomalies [5].

MRI plays a central role in lesion differentiation by enabling comprehensive assessment of lesion laterality, anatomical origin, relationship to adjacent pelvic structures, internal signal characteristics, and associated urinary tract abnormalities [23]. Particular diagnostic value lies in determining whether the lesion is midline, paramedian, or lateralized, as this feature substantially narrows the differential diagnosis.

A lateralized cystic lesion arising from the seminal vesicle in association with ipsilateral renal agenesis is highly suggestive of Zinner syndrome and represents the key imaging pattern that distinguishes it from other retrovesical cystic lesions [5].

## 8. Diagnostic Pitfalls

Several diagnostic pitfalls may complicate the radiologic evaluation of Zinner syndrome and delay the correct diagnosis. A practical understanding of these challenges is essential for both radiologists and clinicians.

One common pitfall is misclassifying the lesion as a Müllerian duct cyst, particularly when lesion laterality is not carefully assessed. Because Müllerian duct cysts are typically midline structures, whereas seminal vesicle cysts are usually lateralized and associated with the ipsilateral seminal vesicle, careful evaluation of the lesion’s exact location and its relationship to adjacent pelvic structures is crucial. This distinction can be improved by multiplanar MRI assessment and by confirming the continuity of the lesion with the seminal vesicle.

Another frequent error is the failure to inspect the renal fossae, which may lead to overlooking the hallmark association of ipsilateral renal agenesis or severe renal dysgenesis. Since this finding is a key component of Zinner syndrome, imaging should systematically include the upper urinary tract, and the renal fossae should always be reviewed when a retrovesical cystic lesion is identified.

A further diagnostic challenge is the under-recognition of vas deferens or ejaculatory duct abnormalities. Incomplete visualization of these structures may prevent identification of ejaculatory duct obstruction, an important component of the syndrome. High-resolution pelvic MRI, especially T2-weighted sequences in multiple planes, can help demonstrate ductal dilatation, atresia, or abnormal insertion.

Additionally, ectopic ureterocele may be confused with a seminal vesicle cyst, especially when imaging fields are incomplete or when the distal ureter is not fully visualized [4,11]. This pitfall can be avoided by carefully tracing the distal ureter and assessing its insertion site, ideally with dedicated urographic sequences or extended field-of-view MRI covering the entire abdominopelvic region.

Another important pitfall is overcalling neoplasms in cysts with proteinaceous or hemorrhagic content. Such cysts may show atypical MRI signal characteristics, including increased T1 signal intensity or apparent internal complexity, potentially mimicking solid lesions or neoplasms [23]. Correlation with diffusion-weighted imaging, post-contrast sequences, and clinical presentation is essential to avoid misinterpretation.

Overall, a systematic imaging approach that includes assessment of lesion laterality, renal fossae, seminal tract anatomy, and distal ureteral course can substantially reduce diagnostic errors and improve confidence in diagnosing Zinner syndrome [28].

## 9. Case Report Presentation

### 9.1. Clinical and Paraclinical Findings

A 29-year-old male presented with a 3-year history of primary infertility in a married couple, associated with chronic pelvic discomfort and rare episodes of dysuria. There was no history of endocrine or systemic disease. Urologic examination was otherwise unremarkable.

Routine hematological and biochemical investigations were within normal limits. Urinalysis and urine culture were negative.

Semen analysis demonstrated low ejaculate volume with severe oligoasthenozoospermia. Semen pH was 7, while fructose levels were not assessed. Sperm DNA fragmentation testing showed an elevated fragmentation index of approximately 25%, supporting a significant male-factor contribution to infertility.

During follow-up, the patient was managed conservatively with regular urological surveillance. Surgical treatment was proposed but declined by the patient. Over more than 6 years of reported infertility symptoms (including the 3 years preceding initial diagnosis), no improvement in fertility parameters or clinical symptoms was documented.

### 9.2. Imaging Findings

The patient was referred for radiologic evaluation because of persistent pelvic discomfort, lower urinary tract symptoms, and infertility.

#### 9.2.1. Abdominal and Pelvic Ultrasonography

Ultrasonography demonstrated absence of the left kidney from the renal fossa, consistent with left renal agenesis, with compensatory hypertrophy of the right kidney.

**Figure 1:**
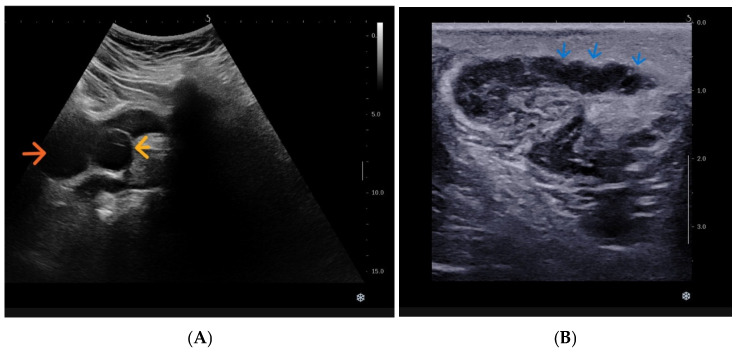
(**A**) Pelvic sonography showing left ureterocele (yellow arrow), protruding into the urinary bladder lumen (orange arrow). (**B**) Dilated left vas deferens (blue arrows).

A well-defined anechoic retrovesical cystic lesion was identified on the left, corresponding to a cystically dilated seminal vesicle. Additionally, a cystic lesion protruding into the urinary bladder lumen at the left vesicoureteric junction was observed, suggestive of ureterocele-like dilatation (Figure 1).

Further abdominal sonographic evaluation demonstrated continuity of this lesion with a tubular cystic structure in the left juxtavesical region, corresponding to a dilated distal blind-ending ureteric remnant.

#### 9.2.2. Scrotal Ultrasonography

Both testes were normal in size, echogenicity, and vascularization. A micronodular dystrophic calcification was noted in the lower third of the right testis.

On the left, markedly dilated supratesticular tubular structures were identified, extending cranially into the inguinal canal and continuing with a dilated ductus deferens, consistent with distal seminal outflow obstruction. The epididymides were otherwise unremarkable, and no intratesticular cystic lesions were detected.

#### 9.2.3. Contrast-Enhanced CT Urography

**Figure 2:**
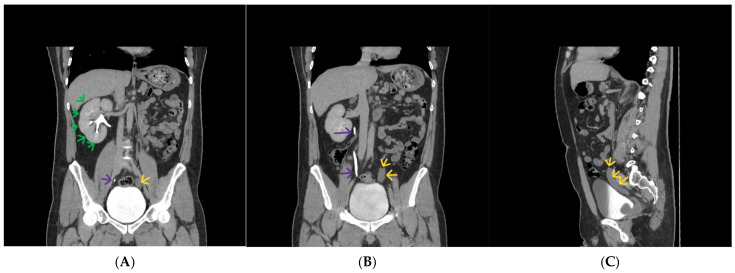
(**A**–**C**) Coronal and sagittal reformatted CT images, delayed phase, showing hypertrophied right kidney (green arrows) and absent left kidney. Left ureter with proximal blind-ending (yellow arrows). Normal right ureter (purple arrows). Normal excretion of contrast by right kidney with accumulation of contrast in urinary bladder.

CT urography confirmed complete left renal agenesis, with no renal parenchyma identified in the left renal fossa (Figure 2).

**Figure 3:**
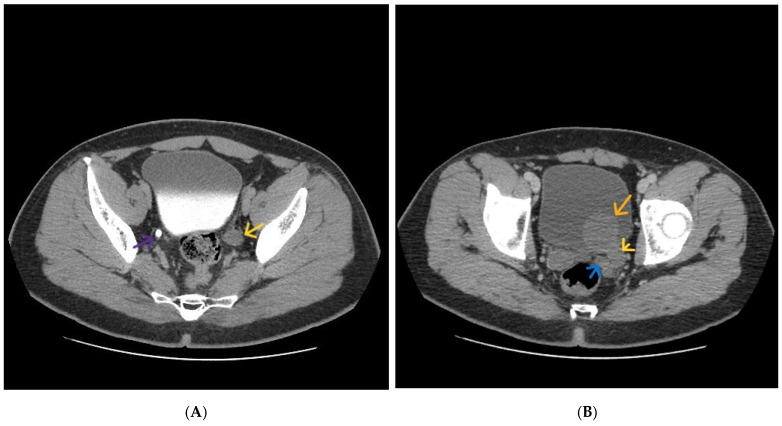
(**A**) axial CT image, delayed phase, showing dilated left ureter (yellow arrow). Normal right ureter (purple arrow). (**B**) axial CT image. Dilated ejaculatory duct (blue arrow). Left ureterocele (orange arrow). Dilated left ureter (yellow arrow).

A tubular cystic structure was seen along the expected course of the left ureter, extending from a blind-ending proximal segment terminating at the level of the L3 vertebral body to the distal juxtavesical region, where it formed a cystic intravesical dilatation with ureterocele-like morphology (Figure 2 and Figure 3).

In the left retrovesical region, a multilobulated cystic lesion corresponding to cystic dilatation of the left seminal vesicle was identified, extending toward the midline and prostatic base, with associated dilatation of the ipsilateral ejaculatory duct.

The right kidney showed compensatory hypertrophy with preserved excretory function. The right ureter and right seminal vesicle were normal. No additional abnormalities of the bladder wall or prostate were identified.

#### 9.2.4. Pelvic Magnetic Resonance Imaging

MRI was performed for further anatomical delineation and tissue characterization.

The examination confirmed cystic dilatation of the left seminal vesicle, associated with ipsilateral dilatation of the vas deferens and ejaculatory duct, representing the typical seminal tract component of Zinner syndrome.

In addition, MRI clearly demonstrated a blind-ending dilated left ureteric remnant extending to an intravesical ureterocele-like cystic dilatation, representing an associated mesonephric duct anomaly and the most atypical anatomic feature in this case.

**Figure 4:**
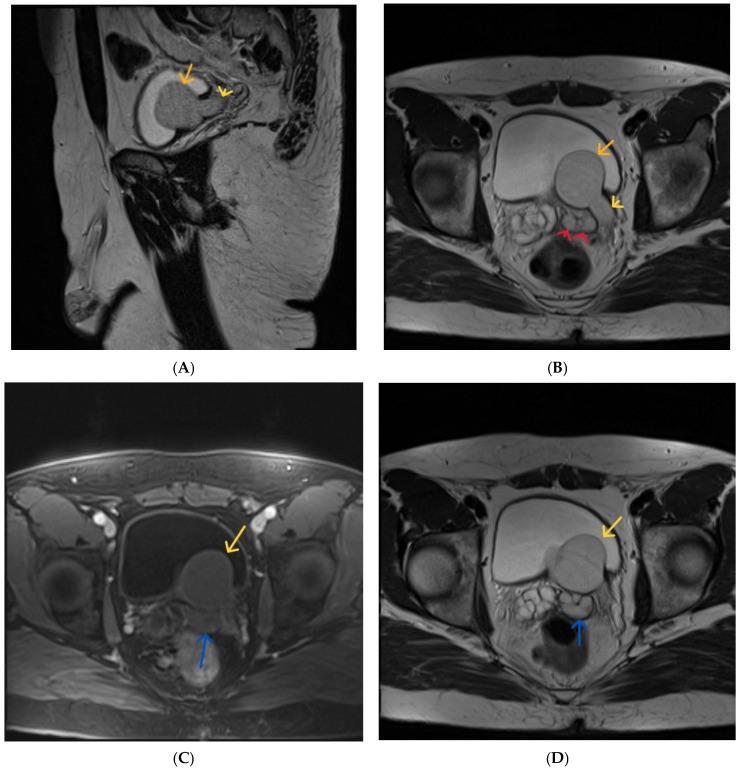

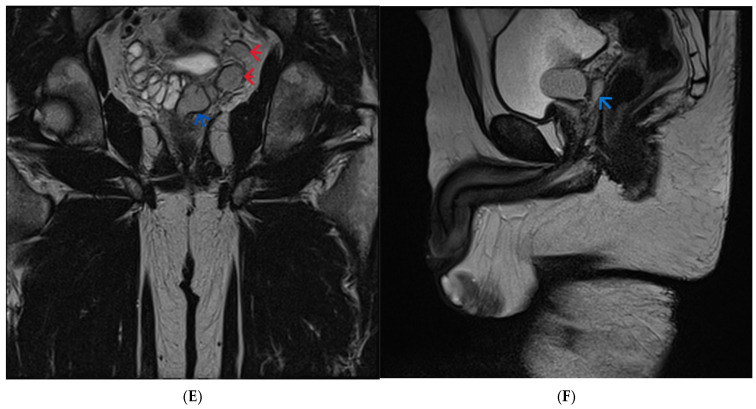
(**A**) sagittal T2-weighted MR image. Left ureterocele (orange arrow), enlarged left ureter (yellow arrow). (**B**) axial T2-weighted MR image. Left ureterocele (orange arrow), enlarged left ureter (yellow arrow). Seminal vesicle cysts (red arrows). (**C**) axial fat-suppressed T1-weighted MR image, with contrast. Left ureterocele (yellow arrow), dilated ejaculatory duct (blue arrow). (**D**) axial T2-weighted MR image showing: left ureterocele (yellow arrow), dilated ejaculatory duct (blue arrow). (**E**) coronal T2-weighted MR image showing: dilated ejaculatory duct (blue arrow) and seminal vesicle cysts (red arrows) (**F**) sagittal T2-weighted MR image showing: dilated ejaculatory duct (blue arrow).

The seminal vesicle cyst and dilated vas deferens showed mild T1 hyperintensity and relatively lower T2 signal compared with urine and the contralateral seminal vesicle, suggesting proteinaceous or hemorrhagic content (Figure 4).

The prostate and urinary bladder were otherwise normal, without wall thickening or intraluminal lesions. Both testes were normal in size, with a small intratesticular hemorrhagic focus on the left.

### 9.3. Diagnostic Considerations and Challenges

This case illustrates the diagnostic complexity of congenital mesonephric duct anomalies presenting with infertility and nonspecific lower urinary tract symptoms.

The typical imaging features of Zinner syndrome were clearly present, namely the classic triad of ipsilateral renal agenesis, ipsilateral seminal vesicle cystic dilatation, and ejaculatory duct/vas deferens dilatation causing distal seminal outflow obstruction.

The most distinctive and original finding in this case was the coexistence of a blind-ending ipsilateral ureteric remnant with distal ureterocele-like intravesical dilatation, representing an associated Wolffian duct malformation beyond the classic triad. This additional anomaly broadens the anatomic spectrum of Zinner syndrome and constitutes the most notable radiologic feature of the present case.

The initial differential diagnosis of the retrovesical cystic lesion included Müllerian duct cyst, prostatic utricle cyst, ejaculatory duct cyst, and ectopic ureterocele. Multimodality imaging was essential for accurate characterization.

Ultrasonography served as the initial screening modality, detecting renal agenesis and the pelvic cystic lesions. CT urography provided precise delineation of the urinary tract anatomy and demonstrated the blind-ending ureteric remnant with ureterocele-like distal dilatation. MRI offered superior soft-tissue characterization, allowing confident identification of seminal vesicle origin and proteinaceous/hemorrhagic content.

The combined findings of ipsilateral renal agenesis, cystic dilatation of the seminal vesicle, ejaculatory duct and vas deferens dilatation, together with a blind-ending ipsilateral ureteric remnant terminating in a ureterocele-like dilatation, supported the diagnosis of Zinner syndrome with associated mesonephric duct anomaly.

## 10. Discussion

Zinner syndrome is a rare congenital mesonephric (Wolffian) duct malformation classically defined by the triad of ipsilateral renal agenesis, seminal vesicle cystic dilatation, and ejaculatory duct obstruction, resulting from abnormal embryologic development of the distal mesonephric duct and ureteric bud [1,4,19]. Because its clinical manifestations are often absent or nonspecific, the diagnosis is usually established radiologically, most commonly in young adults presenting with pelvic discomfort, lower urinary tract symptoms, or infertility [21]. In this context, imaging is not merely confirmatory but central to diagnosis, anatomical characterization, differential diagnosis, and therapeutic planning [19,23].

In the present case, the classical triad was clearly demonstrated. However, the most diagnostically relevant and original finding was the coexistence of a blind-ending ipsilateral ureteric remnant terminating in a ureterocele-like intravesical dilatation, a feature that extends beyond the classical radiologic spectrum of Zinner syndrome and substantially increases the educational value of the case. It is therefore important to distinguish between the typical defining anatomy of Zinner syndrome and the atypical coexisting mesonephric anomaly observed here.

### 10.1. Typical Versus Atypical Anatomy and Diagnostic Relevance

The imaging findings in this case can be analytically divided into a typical and an atypical component. The typical component consisted of left renal agenesis, ipsilateral seminal vesicle cystic dilatation, and associated dilatation of the ejaculatory duct and vas deferens, producing evidence of distal seminal outflow obstruction. These findings correspond closely to the classical radiologic pattern repeatedly described in case reports and small series [4,6,27,28,29].

The atypical component was represented by a tubular blind-ending structure extending along the expected course of the left ureter and terminating distally in a ureterocele-like intravesical cystic dilatation. This finding should not be interpreted as part of the classical triad itself, but rather as a coexisting associated mesonephric duct anomaly. Embryologically, this association remains coherent, as the ureteric bud arises from the mesonephric duct, and abnormal separation or incomplete involution may result in persistence of a blind-ending ureteric remnant despite ipsilateral renal agenesis [1,11,19].

This distinction is particularly important because the ureterocele-like component may create diagnostic ambiguity. Without careful radiologic correlation, it may be mistaken for an isolated ectopic ureterocele, distal ectopic ureter, or another bladder-adjacent cystic lesion unrelated to Zinner syndrome. The present case therefore expands the radiologic spectrum of mesonephric duct anomalies associated with the syndrome and highlights an uncommon but clinically relevant diagnostic pitfall.

### 10.2. Practical Contribution of Each Imaging Modality in This Specific Case

The diagnosis in this case was established through sequential multimodality imaging correlation, with each modality contributing a distinct and complementary role.

Ultrasonography provided the initial diagnostic suspicion by demonstrating absence of the left kidney, a lateralized retrovesical cystic lesion, and an intravesical cystic protrusion suggestive of ureterocele-like dilatation. Its principal contribution was therefore syndrome recognition rather than complete characterization. In particular, the combination of unilateral renal agenesis and a retrovesical cyst in a young infertile male should immediately raise suspicion for Zinner syndrome [21,30].

CT urography played a decisive role in clarifying the urinary tract anatomy. In this case, CT demonstrated the blind-ending tubular structure following the expected left ureteric course and confirmed its continuity with the distal intravesical ureterocele-like dilatation. This was essential in distinguishing the lesion from an isolated pelvic cyst and in establishing its origin as a persistent ureteric remnant [22,31].

MRI provided the final diagnostic resolution by confirming the seminal vesicle origin of the retrovesical cystic lesion, demonstrating associated ejaculatory duct and vas deferens dilatation, and characterizing the proteinaceous or hemorrhagic content through signal characteristics. Most importantly, MRI allowed clear separation of the seminal tract anomaly from the adjacent blind-ending ureteric remnant, thereby resolving the principal diagnostic uncertainty [23,32].

Thus, the imaging pathway in this case followed a practical and clinically relevant sequence: ultrasound raised suspicion, CT defined anatomy, and MRI established definitive tissue characterization and lesion origin.

### 10.3. Main Diagnostic Challenge and Imaging Discriminators

The main diagnostic challenge in this case was the differentiation of the lateralized seminal vesicle cyst from other retrovesical cystic lesions, particularly in the setting of associated distal urinary tract abnormalities.

The principal differential diagnoses include Müllerian duct cyst, prostatic utricle cyst, ejaculatory duct cyst, ectopic ureterocele, and distal ectopic ureteric remnant cyst. Among these, the most important imaging discriminator remains the combination of lesion lateralization and ipsilateral renal agenesis.

Unlike Müllerian duct cysts and prostatic utricle cysts, which are typically midline, seminal vesicle cysts are classically paramedian or lateralized and located posterior to the bladder [11,19]. The presence of ipsilateral renal agenesis remains the strongest radiologic sign supporting the diagnosis of Zinner syndrome and should always prompt directed evaluation of the seminal vesicles and ejaculatory ducts [1,4,28].

In the present case, the blind-ending ureteric remnant increased the complexity of the differential diagnosis by mimicking a separate distal urinary tract lesion. Recognition of its tubular course and embryologic coherence with Wolffian duct maldevelopment was therefore critical for correct interpretation.

### 10.4. Comparison with Published Cases and Contribution to Literature

The present case shares the core radiologic features consistently described in previously published reports of Zinner syndrome, namely unilateral renal agenesis, ipsilateral seminal vesicle cystic dilatation, and ejaculatory duct obstruction. Similar imaging patterns have been reported by Hofmann et al., Karki et al., Boui et al., and Knutsen et al., particularly in young adult men presenting with infertility and pelvic symptoms [1,4,6,29]. Likewise, the MRI-based series by Elsorougy et al. emphasized the value of pelvic MRI in delineating seminal vesicle cysts and ejaculatory duct anomalies, findings that are central to the present case [23].

What distinguishes the current case from the majority of published reports is the presence of the blind-ending ipsilateral ureteric remnant with distal ureterocele-like intravesical dilatation, which appears distinctly uncommon in the literature. While several reports describe associated Wolffian duct variants, including ectopic seminal vesicle position, unilateral testicular agenesis, and aberrant vascular anatomy [27,33,34], the specific association observed here is rarely emphasized.

This feature is particularly important because it may initially redirect the radiologic interpretation toward ectopic ureterocele or another bladder-adjacent cystic lesion. By demonstrating the coexistence of a seminal vesicle cyst with a persistent ureteric remnant, the present case broadens the currently recognized imaging spectrum of Zinner syndrome and reinforces the concept of a wider continuum of mesonephric duct anomalies.

Accordingly, the main contribution of this report lies not simply in illustrating the classical triad but in documenting a rare associated anatomic variant with direct implications for differential diagnosis and radiologic reporting.

### 10.5. Clinical Implications: Psychological Symptoms and Impact of Treatment

Although the primary focus of this report is radiologic, the diagnosis has important clinical implications that extend beyond anatomical characterization, particularly in patients presenting during their reproductive years.

Implications of infertility: infertility remains one of the most significant clinical consequences of Zinner syndrome and is most commonly related to ejaculatory duct obstruction and impaired sperm transport secondary to seminal tract anomalies [28,29]. In the present case, the imaging findings directly correlated with severe semen abnormalities and persistent primary infertility. Accurate radiologic reporting of the extent of ejaculatory duct and vas deferens involvement is therefore essential for fertility counseling and consideration of assisted reproductive strategies.

Emotional responses: the diagnosis of a congenital anomaly associated with infertility may lead to significant psychological distress, including anxiety, uncertainty regarding reproductive potential, reduced self-esteem, and concerns related to masculinity and long-term sexual health [25,35]. Chronic pelvic discomfort may further contribute to emotional burden and diminished quality of life.

Sexual function: sexual function may be affected both by the underlying obstructive anatomy and by associated psychological stress [36]. Patients may experience painful ejaculation, dysejaculation, or secondary sexual dysfunction [35]. In cases requiring intervention, concerns regarding postoperative ejaculatory changes and persistent subfertility should also be addressed [20,37].

Family dynamics: the effects of infertility frequently extend to partner relationships and family expectations, particularly in cultural contexts where parenthood carries substantial social significance. These considerations support a multidisciplinary management approach involving radiology, urology, reproductive medicine, and counseling when appropriate.

### 10.6. Practical Future Priorities

Future priorities should focus on improved radiologic awareness, more structured reporting, and greater consistency in MRI-based evaluation of male pelvic cystic lesions.

In practical terms, radiology reports should systematically address the presence or absence of the ipsilateral kidney, lesion lateralization, seminal vesicle origin, ejaculatory duct involvement, vas deferens dilatation, and any associated ureteric remnants or ureterocele-like anomalies. Greater familiarity with these imaging patterns may improve diagnostic confidence and reduce confusion with retrovesical mimics [19,23].

## 11. Teaching Point and Conclusions

Zinner syndrome remains an uncommon and frequently underrecognized entity, largely owing to its variable clinical presentation and the nonspecific nature of the associated symptoms. In many cases, the diagnosis is established incidentally during imaging studies performed for unrelated indications.

Imaging plays a central role in both diagnosis and clinical management. Ultrasound is commonly used as the initial imaging examination, allowing detection of renal agenesis and associated cystic pelvic lesions; however, it is limited in terms of detailed anatomical assessment. Computed tomography provides a comprehensive overview of urinary tract anatomy and associated anomalies, whereas magnetic resonance imaging offers superior soft-tissue contrast and multiplanar evaluation of seminal vesicle pathology and ejaculatory duct involvement. MRI is particularly valuable for lesion characterization and problem-solving, enabling confident differentiation from other cystic pelvic entities. Recognition of the characteristic constellation of findings, supported by embryologic correlation, is essential to avoid diagnostic pitfalls and unnecessary invasive procedures.

The presented clinical case further illustrates the potential diagnostic challenges associated with atypical presentations and overlapping imaging features. Accurate radiologic identification allows appropriate urologic referral, fertility counseling, and tailored management, ranging from conservative follow-up in asymptomatic patients to minimally invasive intervention in symptomatic cases. Increased awareness among radiologists is therefore essential for timely diagnosis and optimized patient care.

## 12. Take-Home Messages for Radiologists

Consider Zinner syndrome in male patients presenting with a pelvic cystic lesion associated with ipsilateral renal agenesis.

Interpret male pelvic cysts within an embryologic context to differentiate them from Müllerian duct and prostatic utricle cysts.

As many patients are asymptomatic, radiologists are often the first to suggest the diagnosis.

MRI is the preferred problem-solving modality for detailed assessment of seminal vesicle cysts and ejaculatory duct involvement.

Early recognition facilitates timely urologic referral, fertility counseling, and helps avoid unnecessary investigations or interventions.

## Figures and Tables

**Table 1:** Practical MRI Approach for Zinner syndrome.

Step	Objective	Key MRI Findings	Practical Tip
1	Confirm the diagnostic triad	Seminal vesicle cyst (paramedian, posterior to bladder); ipsilateral renal agenesis; ejaculatory duct obstruction	Always evaluate the upper abdomen to confirm absence of the ipsilateral kidney
2	Define lesion origin	Elongated or lobulated cyst; continuity with seminal vesicle; separate from midline prostate	Paramedian location favors seminal vesicle origin over midline cysts
3	Assess cyst content	T2 hyperintense (simple fluid); T1 hyperintense (proteinaceous or hemorrhagic); internal debris in complex lesions	Add fat-suppressed T1-weighted imaging if content is indeterminate
4	Evaluate complications	Wall thickening or enhancement; restricted diffusion; mass effect on adjacent organs	Use DWI and contrast-enhanced imaging only when complexity or infection is suspected

**Table 2:** Recommended MRI Protocol (Minimum Practical Set).

Category	Sequence	Purpose	Practical Notes
Core	Axial T2-weighted	Primary anatomical assessment	Best sequence for identifying seminal vesicle cyst and local anatomy
Core	Sagittal T2-weighted	Assessment of midline structures and relationships	Useful for evaluating bladder, prostate, and rectum
Core	Coronal T2-weighted	Evaluation of lesion extent and symmetry	Helps confirm unilateral involvement
Core	Axial T1-weighted	Baseline tissue characterization	Detects intrinsic T1 hyperintensity (hemorrhage or proteinaceous content)
Problem-solving	Fat-suppressed T1-weighted	Confirmation of hemorrhagic or proteinaceous content	Recommended when T1 signal is high or equivocal
Problem-solving	Diffusion-weighted imaging (DWI)	Detection of infection or complex cysts	Restricted diffusion suggests abscess or infection
Problem-solving	Post-contrast T1-weighted	Evaluation of enhancement patterns	Reserved for suspected infection, solid components, or atypical findings

**Table 3:** Radiologic Differential Diagnosis of Seminal Vesicle Cysts and Other Male Pelvic Cystic Lesions.

Lesion	Typical Location	Laterality	Ultrasound (US)	CT Findings	MRI Signal Characteristics	Key Radiologic Features	Associated Findings	Key Discriminators from Zinner Syndrome
Seminal vesicle cyst	Postero- lateral to bladder, superior to prostate	Usually, unilateral	Anechoic or hypoechoic cyst; posterior acoustic enhancement	Well-defined low- attenuation cyst; may contain hyperdense material if hemorrhagic	T1: Variable T2: Hyperintense	Continuity with seminal vesicle; asymmetric seminal vesicles	Ipsilateral renal agenesis or dysplasia (Zinner syndrome)	No renal agenesis
Müllerian duct cyst	Midline between bladder and rectum	Midline	Anechoic midline cyst	Low-attenuation midline cyst	T1: Hypointense T2: Hyperintense	No communication with seminal vesicles	No renal anomalies	Cyst location: midline No renal agenesis
Prostatic utricle cyst	Midline, intraprostatic	Midline	Small midline cyst within prostate	Small low-density cyst	T1: Hypointense T2: Hyperintense	Communicates with prostatic urethra; small size	Hypospadias, intersex anomalies	Cyst location: midline No renal agenesis
Ejaculatory duct cyst	Paramedian, near verumontanum	Paramedian or bilateral	Cystic tubular structure near midline	Tubular low-density lesion	T1: Low–intermediate T2: Hyperintense	Upstream dilatation of seminal vesicles	Infertility, ejaculatory dysfunction	similar location, but no renal or seminal vesicles agenesis
Vas deferens cyst	Along vas deferens course	Unilateral	Tubular anechoic structure	Tubular low-attenuation lesion	T1: Low T2: Hyperintense	Follows vas deferens anatomy	Rare	Not centered in seminal vesicle region. No renal agenesis
Bladder diverticulum	Bladder wall	Variable	Cystic outpouching communicating with bladder	Contrast-filled outpouching	Urine signal on all sequences	Size varies with bladder filling	Bladder outlet obstruction	Communicates with bladder lumen and changes size with voiding
Ureterocele (ectopic)	Bladder base	Usually unilateral	Cystic lesion within bladder lumen	“Cyst-within-a-cyst” appearance	T1: Low T2: Hyperintense	Typical intravesical location	Duplex collecting system	associated with duplicated collecting system; shows ureteral connection
Tailgut/duplication cyst	Presacral, posterior to rectum	Midline or paramedian	Multiloculated cystic mass	Low-attenuation presacral lesion	T1: Variable T2: Hyperintense	No urogenital tract communication	GI embryologic origin	Location: retrorectal. No urogenital duct connection.
Infected cyst/ abscess	Variable	Variable	Complex cyst with internal echoes	Thick-walled lesion with enhancement	T1/T2: Variable; restricted diffusion	Peripheral enhancement, diffusion restriction	Fever, elevated inflammatory markers	Clinical and biological inflammatory changes.

## Data Availability

No new data were created or analyzed in this study. Data sharing is not applicable to this article.
